# Inhibition of galectin‐3 augments the antitumor efficacy of PD‐L1 blockade in non‐small‐cell lung cancer

**DOI:** 10.1002/2211-5463.13088

**Published:** 2021-01-31

**Authors:** Hongxin Zhang, Pengfei Liu, Yan Zhang, Lujun Han, Zhihui Hu, Ziqi Cai, Jianhui Cai

**Affiliations:** ^1^ Department of Surgery Hebei Medical University Shijiazhuang China; ^2^ Department of Oncology Tianjin Academy of Traditional Chinese Medicine Affiliated Hospital China; ^3^ Department of Oncology Shijiazhuang First Hospital China; ^4^ Hebei Engineering Technology Research Center for Cell Therapy Hebei HOFOY Bio‐Tech Co. Ltd Shijiazhuang China; ^5^ Department of Surgery Department of Oncology & Immunotherapy Hebei General Hospital Shijiazhuang China

**Keywords:** galectin‐3, immunotherapy, lung cancer, NSCLC, PD‐L1, tumor

## Abstract

Multiple clinical trials have shown that monoclonal antibodies (mAbs) against programmed death‐ligand 1 (PD‐1/PD‐L1) can benefit patients with lung cancer by increasing their progression‐free survival and overall survival. However, a significant proportion of patients do not respond to anti‐PD‐1/PD‐L1 mAbs. In the present study, we investigated whether galectin (Gal)‐3 inhibitors can enhance the antitumor effect of PD‐L1 blockade. Using the NSCLC‐derived cell line A549, we examined the expression of Gal‐3 in lung cancer cells under hypoxic conditions and investigated the regulatory effect of Gal‐3 on PD‐L1 expression, which is mediated by the STAT3 pathway. We also explored whether Gal‐3 inhibition can facilitate the cytotoxic effect of T cells induced by PD‐L1 blockade. The effects of combined use of a Gal‐3 inhibitor and PD‐L1 blockade on tumor growth and T‐cell function were also investigated, and we found that hypoxia increased the expression and secretion of Gal‐3 by lung cancer cells. Gal‐3 increased PD‐L1 expression via the upregulation of STAT3 phosphorylation, and administration of a Gal‐3 inhibitor enhanced the effect of PD‐L1 blockade on the cytotoxic activity of T cells against cancer cells *in vitro*. In a mouse xenograft model, the combination of a Gal‐3 inhibitor and PD‐L1 blockade synergistically suppressed tumor growth. Furthermore, the administration of a Gal‐3 inhibitor enhanced T‐cell infiltration and granzyme B release in tumors. Collectively, our results show that Gal‐3 increases PD‐L1 expression in lung cancer cells and that the administration of a Gal‐3 inhibitor as an adjuvant enhanced the antitumor activity of PD‐L1 blockade.

## Graphical abstract

Galectin (Gal)‐3 is involved in the immune response, and highly overexpressed and secreted by lung cancer cells. This study showed that the increased expression of Gal‐3 was induced by hypoxia, which then contributed to immune suppression through the STAT3 pathway in lung cancer. Furthermore, a Gal‐3 inhibitor enhanced the antitumor effect by PD‐L1 blockade through promoting T‐cell infiltration in lung cancer.

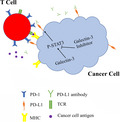

**Abbreviations**
CI: combination index
E:T: effector‐to‐target
ELISA: Enzyme‐linked immunosorbent assay
Gals: Galectins
ICIs: immune checkpoint inhibitors
IHC: immunochemistry
mAbs: monoclonal antibodies
PBMCs: peripheral blood mononuclear cells
PD‐1: programmed death receptor
SD: standard error
TILs: tumor‐infiltrating lymphocytes

Lung cancer is the leading cause of cancer‐related death worldwide. In 2018, the incidence and mortality of lung cancer were estimated to be 11.6% and 18.4%, respectively [1]. In recent years, to improve the prognosis of patients with lung cancer, immunotherapy has been introduced into clinical trials. Immunotherapy is known to activate cytotoxic T lymphocytes (also known as CD8+ T cells or killer T cells) in patients so that they recognize and kill cancer cells more effectively. As one type of immunotherapy strategy, immune checkpoint inhibitors (ICIs) have been developed to stimulate the immune system by blocking inhibitory immune checkpoint molecules. ICIs then promote immune‐mediated elimination of tumor cells, such as through antibodies that target programmed death receptor 1 (PD‐1). PD‐1 and its ligand play an important role in the antitumor immune response by inhibiting T‐cell activation, cytokine production, and cytolytic function, while anti‐PD‐1/PD‐L1 monoclonal antibodies (mAbs) inhibit the interaction between the inhibitory receptor on T cells and its ligands on cancer cells, thereby reestablishing T‐cell reactivity and antitumor effector functions [2]. Multiple clinical trials have shown that anti‐PD‐1/PD‐L1 mAbs can benefit patients with lung cancer by increasing their progression‐free survival and overall survival [3, 4]. However, a significant proportion of patients do not respond to anti‐PD‐1/PD‐L1 mAbs. One of the key reasons for their nonresponse is because tumors can induce immunosuppression via multiple pathways [5, 6, 7]. For example, cancer cells can contribute to immunosuppression in tumors by secreting cytokines that act as inhibitory factors against T‐cell infiltration [8].

Galectins (Gals) belong to a family of β‐galactoside‐binding lectins with evolutionarily conserved carbohydrate‐recognition domains; thus far, 15 Gals have been identified in vertebrates and are found to be expressed in various tissues and organs [9]. Human Gal‐3 is a 35‐kDa chimera‐type Gal that is encoded by the LGALS3 gene on chromosome 14. Gal‐3 may be expressed in the cytoplasm, nucleus, or the cellular microenvironment [10, 11]. Gal‐3 has also been shown to be involved in the immune response. For example, Gal‐3 can regulate the functions of dendritic cells. Chung et al found that Gal‐3 can inhibit the differentiation of human monocytes into DCs *in vitro* [12] and that blocking Gal‐3 can inhibit the expression of proinflammatory cytokines, such as IL‐6 and IL‐1β, and can upregulate the expression of IL‐10 and IL‐12 in human monocyte‐derived DCs [13]. In Gal‐3‐deficient mice, DCs produced significantly higher levels of cytokines related to the IL‐23/IL‐17 axis and lower levels of IL‐12 and IFN‐γ [14]. Additionally, Gal‐3 plays a crucial role in promoting tumor‐driven immune suppression, which can suppress the expansion of tumor‐reactive T cells [15]. Moreover, Gal‐3 is highly overexpressed and secreted into the surrounding microenvironment by lung cancer cells, which may be related to cancer progression [15, 16, 17]. Therefore, we speculated that Gal‐3 might regulate PD‐L1 expression, which could then contribute to immune suppression in lung cancer. The inhibition of Gal‐3 as an adjuvant approach could eliminate immunotherapy resistance in tumors and thus enhance the antitumor effects of anti‐PD‐1/PD‐L1 mAbs. Thus, in the present study, we investigated the regulatory effect of Gal‐3 on PD‐L1 expression and the potential pathways through which it functions in the NSCLC cell line A549, and we also examined the effects of combined treatment using a Gal‐3 inhibitor with PD‐L1 blockade *in vitro* and *in vivo*.

## Materials and methods

### Cell culture

The human lung adenocarcinoma cell line A549 was donated by the Hebei Institute of Oncology. A549 cells were maintained in RPMI‐1640 (Gibco, Gaithersburg, MD, USA) supplemented with penicillin, streptomycin, and 10% fetal calf serum (Gibco) and were cultured at 37 °C in a humidified atmosphere of 5% CO_2_.

To establish hypoxic conditions, cells were maintained in culture medium in a chamber with 5% CO_2_, 95% N_2_, and 0.5% O_2_ at 37 °C for 24 h.

For the *in vitro* experiments, the cells were treated with Gal‐3 (purchased from Sigma, St. Louis, CA, USA; dissolved in saline) at a concentration of 5 μg·mL^−1^ [18] and a Gal‐3 inhibitor (GB1107, purchased from MedChem Express, Monmouth Junction, NJ, USA) at a concentration of 1 μm in DMSO [19] (see IC50 assay data in Fig. S1).

### SiRNA transfection

Cells were seeded in 12‐well culture plates (10^5^ cells/well) and were then transfected with 40 nm anti‐STAT3 siRNA or a scrambled probe (Santa Cruz, Dallas, CA, USA) using Lipofectamine 2000 (Invitrogen, Carlsbad, CA, USA) according to the manufacturer's instructions. Twenty‐four hours after transfection, the cells were used in other experiments, and western blotting was performed to validate the results of STAT3 inhibition.

### Western blotting

Cell pellets were lysed in RIPA buffer containing proteinase inhibitor. Equal amounts of protein (20 μg) were loaded on 8–10% gels and subjected to SDS/PAGE and then electrotransferred onto Polyvinylidene fluoride membranes (Millipore, Burlington, MA, USA). The membranes were then blocked with BSA and incubated with the appropriate primary antibody (1 : 3000) overnight at 4 °C, as indicated in the manufacturer's protocol. Subsequently, the membrane was incubated with secondary antibodies (1 : 3000, anti‐rabbit IgG or anti‐mouse IgG; Abcam, Cambridge, UK). Next, the protein level on the blot was detected using a Western Bright ECL kit (Bio‐Rad Laboratories, Hercules, CA, USA). Equal loading of the sample was validated by the detection of β‐actin. The following antibodies were used in this study: anti‐PD‐L1 (rabbit, monoclonal, ab213480; Abcam), anti‐STAT3 (rabbit, monoclonal; 30835; Cell Signaling, Danvers, MA, USA), anti‐phospho‐STAT3 (Tyr705, rabbit, monoclonal; 9145; Cell Signaling), and anti‐β‐actin (mouse, monoclonal; sc‐47778; Santa Cruz).

### PBMC preparation

This study was approved by the institutional review board of Hebei Medical University (No. 2020055), which was conformed to the standards set by the Declaration of Helsinki. Written informed consent was obtained from all donors for their participation and publication of their data in accordance with the guidelines verified and approved by Hebei Medical University. Peripheral blood samples were obtained from Hebei Blood Center (Shijiazhuang, China). Then, peripheral blood mononuclear cells (PBMCs) were obtained by the attachment method and were allowed to adhere to plastic culture dishes for 2 h at 37 °C. Next, the attached cells were cultured in RPMI 1640 supplemented with 10% FBS, 10 ng·mL^−1^ lipopolysaccharide (Beyotime, Shanghai, China), 50 ng·mL^−1^ human recombinant granulocyte‐macrophage colony‐stimulating factor (GM‐CSF; Pepro Tech, Rocky Hill, NJ, USA), 20 ng·mL^−1^ human recombinant IL‐4 (PeproTech), 100 U·mL^−1^ recombinant human IL‐2 (PeproTech), 2 mm l‐glutamine, and 50 mm 2‐mercaptoethanol. After 6 days of culture, tumor cell antigen lysates (equivalent to 1 × 10^7^ tumor cells per mL) were added, after which the cells were cultured for another 24 h.

In this study, some experimental groups were treated with a Gal‐3 inhibitor at a concentration of 1 μm in DMSO or atezolizumab at a concentration of 100 nm (InvivoGen, San Diego, CA, USA).

### Tumor antigen preparation

Tumor antigens were prepared by the freeze‐thaw method. Briefly, A549 cells were collected, washed in PBS, resuspended in PBS at a density of 1 × 10^7^ cells per mL, and then stored in a tube. Thereafter, the cells were slowly immersed in liquid nitrogen for 10 min and then immediately placed in a water bath at 37 °C for 10 min. The freeze–thaw procedure was repeated three times, and the sample was then centrifuged at 1700 ***g*** for 10 min. The supernatants were collected as tumor antigen lysates, which were stored at −80 °C until further use.

### Cytotoxicity assay

A 4‐h 51Cr‐release assay was used to evaluate the cytotoxic activity of T cells (isolated from PBMCs) against A549 cells. Briefly, A549 cells (target cells) were seeded in 96‐well plates at a density of 5000 cells per well and were then incubated with ^51^Cr (1 μCi·mL^−1^) overnight. Then, the wells were washed with PBS. Thereafter, PBMCs were added to the wells at effector‐to‐target (E:T) ratios of 5 : 1, 10 : 1, or 20 : 1 in the presence or absence of PD‐L1 blockade and/or a Gal‐3 inhibitor. After 8 h of incubation, the supernatants were collected and analyzed in a gamma counter. Specific lysis was evaluated according to the following formula [20]:

$$\begin{array}{c} \%\;\text{specific release}=[(\text{experimental release}‐\text{spontaneous release})/\\ (\text{maximum release}‐\text{spontaneous release})]\times 100 \end{array}$$

### Mouse xenograft model treatment

All animal experiments were approved by the Committee of Ethics of Hebei Medical University Animal Care and Use and were performed according to the ‘ARRIVE’ guidelines. BALB/c nu/nu nude mice (8 weeks of age, male : female = 1 : 1) were purchased from the Institute of Laboratory Animal Science, the Chinese Academy of Medical Sciences (Beijing, China), and housed in our experimental animal facility (five mice per cage) under standard laboratory conditions with free access to food and water. Each mouse was given subcutaneous (s.c.) injections in the flank region of 1 × 10^6^ cells suspended in 100 μL of PBS mixed with 100 μL of Matrigel (BD Biosciences, San Jose, CA, USA).

Briefly, when the tumor volumes reached 100 mm^3^, all mice received PBMCs via tail vein injection (1 × 10^7^ cells) on days 1 and 8; the following groups were randomly established (eight mice/group): (a) placebo (control); (b) Gal‐3 inhibitor (GB1107); (c) PD‐L1 blockade; and (d) Gal‐3 inhibitor + PD‐L1 blockade. After 28 days of treatment, the mice were euthanized by CO_2_ inhalation (30% of the chamber volume/min).

The anti‐PD‐L1 antibody (rat anti‐PD‐L1, αPD‐L1, clone 10F.9G2; BioLegend, San Diego, CA, USA) used for the *in vivo* blockade experiments was purchased from Bio X Cell. A total of 200 μg of the antibody in PBS was administered twice weekly by intraperitoneal injection, while the Gal‐3 inhibitor (GB1107) was administered at a dose of 10 mg·kg^−1^·day^−1^ by intraperitoneal injection.

Tumor volumes were determined using the following formula: length × width × height × 0.5. All tumors were measured every 2 days using a dial caliper. The tumor growth inhibition index was defined as follows: TGI = (1 – (mean volume of treated tumors)/(mean volume of control tumors)) × 100% [21]. The combination index (CI) of the two therapies was assessed according to the following formula: CI = TGI_A + B_/[TGI_A _+ (1‐ TGI_A_) TGI_B_], where TGI_A+B_ is the inhibition rate of the combination therapy, TGI_A_ is the inhibition rate of the Gal‐3 inhibitor, and TGI_B_ is the inhibition rate of the PD‐L1 blockade. A CI < 0.9 indicates synergism, a CI > 1.1 indicates antagonism, and a CI = 0.9–1.1 indicates an additive relationship [22].

### Histological evaluation and immunochemistry (IHC)

The tumor tissue was fixed, embedded in paraffin, and cut into 5‐μm‐thick sections, which were placed onto glass slides. The sections were then deparaffinized, rehydrated, and subjected to antigen retrieval. Next, the slides were blocked with goat serum and incubated with rabbit mAbs (1 : 200 dilution) against CD3 (ab135372; Abcam) or granzyme and then with a horseradish peroxidase‐labeled anti‐rabbit antibody (46890; Cell Signaling). Finally, the slides were developed with diaminobenzidine and counterstained in hematoxylin. Target protein expression was scored semiquantitatively according to a routine IHC grading system, and the values were used for statistical analysis.

### Enzyme‐linked immunosorbent assay (ELISA)

Cell culture supernatants were collected from cells (1 × 10^7^ cells) that received different treatments. Next, Gal‐3 was detected in the supernatants using an ELISA kit (R&D Systems, Minneapolis, MN, USA) according to the manufacturer's instructions. Briefly, biotinylated antibodies were added to the plate, after which the culture supernatants were added to the wells. Standard reagents were diluted and added to the wells to generate a standard curve. After 2 h of incubation at room temperature, the absorbance was read at 450 nm using a multimode plate reader.

### Statistical analysis

The statistical analysis was performed using the r (R Core Team, https://www.r‑project.org/, New Zealand) environment for statistical computing and graphics (version 3.6.3). Data are expressed as the mean ± standard error (SE). A two‐tailed *t*‐test and ANOVA were used for the statistical analysis. *P* values < 0.05 were considered significant.

## Results

### Hypoxia increased the secretion of Gal‐3 by lung cancer cells

**Fig. 1:**
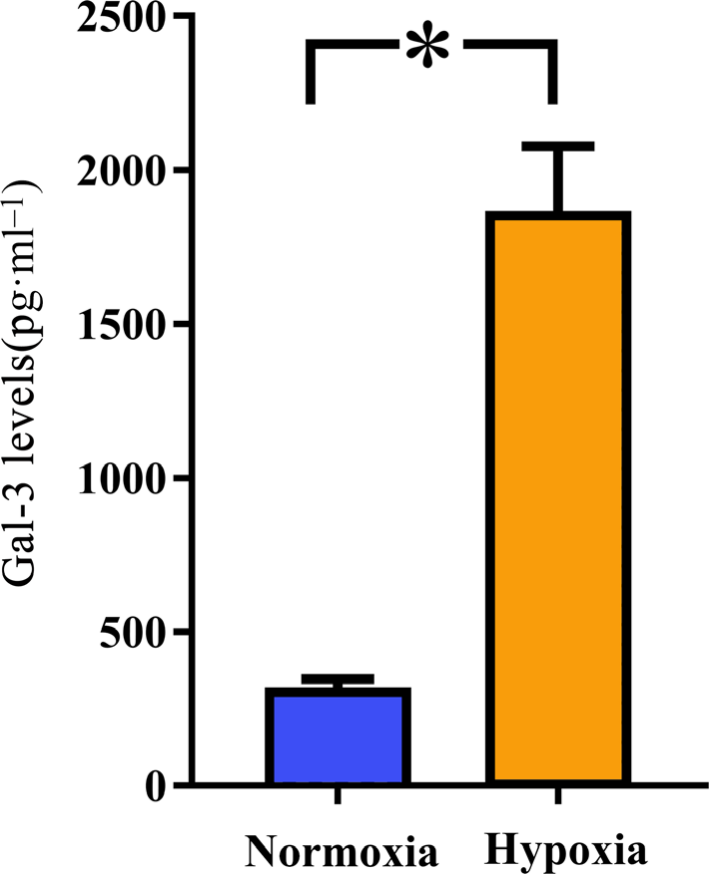
Hypoxia promotes the expression and secretion of Gal‐3 by lung cancer cells. A549 lung cancer cells were cultured under hypoxic or normoxic conditions for 24 h, as described in the Materials and methods. Then, the cell culture medium was collected, and the levels of Gal‐3 were evaluated by ELISA. The results showed that hypoxia significantly increased the expression and secretion of Gal‐3. All experiments were performed in triplicate, and the data are shown as the mean ± SEM. (*P = 0.000, by two‐tailed *t*‐test).

It is well known that hypoxia is a potent microenvironmental factor that promotes the metastatic progression of cancer, local immune suppression, and inhibition of immune killing functions [23, 24]. Thus, we cultured A549 lung cancer cells under hypoxic conditions for 24 h, and our data showed that the Gal‐3 level secreted by A549 cells under hypoxic conditions was significantly increased compared with that secreted by A549 cells under normoxic conditions (Fig. 1).

### Gal‐3 regulated PD‐L1 expression through STAT3 phosphorylation in lung cancer cells

**Fig. 2:**
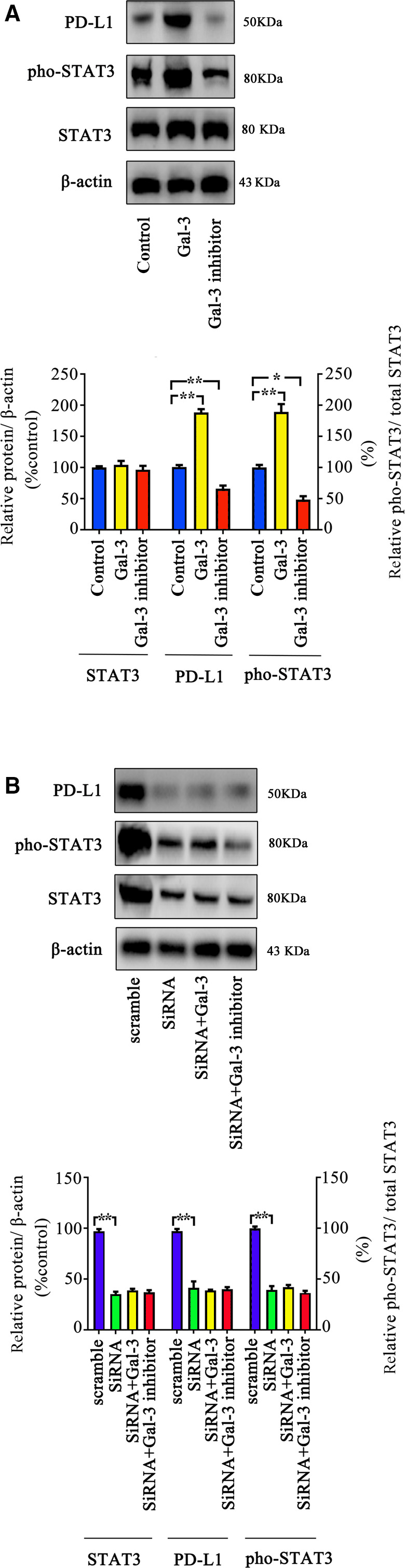
Gal‐3 regulates PD‐L1 expression through the STAT3 pathway. (A) A549 cells were treated with Gal‐3 or a Gal‐3 inhibitor for 48 h, and p‐STAT3, STAT3, and PD‐L1 expression was measured by western blot. The results showed that Gal‐3 can increase the phosphorylation level of STAT3 as well as the PD‐L1 level and that a Gal‐3 inhibitor has the opposite effects. (B) Gal‐3 failed to regulate PD‐L1 expression when STAT3 was knocked down. We used siRNA to knock down STAT3 expression and then treated the cells with Gal‐3 or a Gal‐3 inhibitor for 48 h. The expression of p‐STAT3, STAT3, and PD‐L1 was then evaluated by western blot. All experiments were performed in triplicate, and the data are shown as the mean ± SEM. (*P < 0.05; **P < 0.01, by one‐way ANOVA).

PD‐L1 is a classic immunosuppressive protein located on the cell surface [25]. Therefore, we investigated whether Gal‐3 can impact the expression of PD‐L1 on A549 cells. Treatment with Gal‐3 increased the expression of PD‐L1 in A549 lung cancer cells as well as STAT3 phosphorylation, whereas blocking Gal‐3 with an inhibitor decreased both the expression of PD‐L1 and STAT3 phosphorylation in these cells (Fig. 2A).

To further explore the potential regulatory effect of Gal‐3 on PD‐L1 expression, we knocked down STAT3 expression by siRNA and found that the inhibition of STAT3 pathway activity decreased PD‐L1 expression in A549 cells. Moreover, the administration of Gal‐3 or a Gal‐3 inhibitor failed to regulate PD‐L1 expression when the STAT3 pathway was blocked (Fig. 2B).

### Inhibition of Gal‐3 enhanced cytotoxic T‐cell effector function

**Fig. 3:**
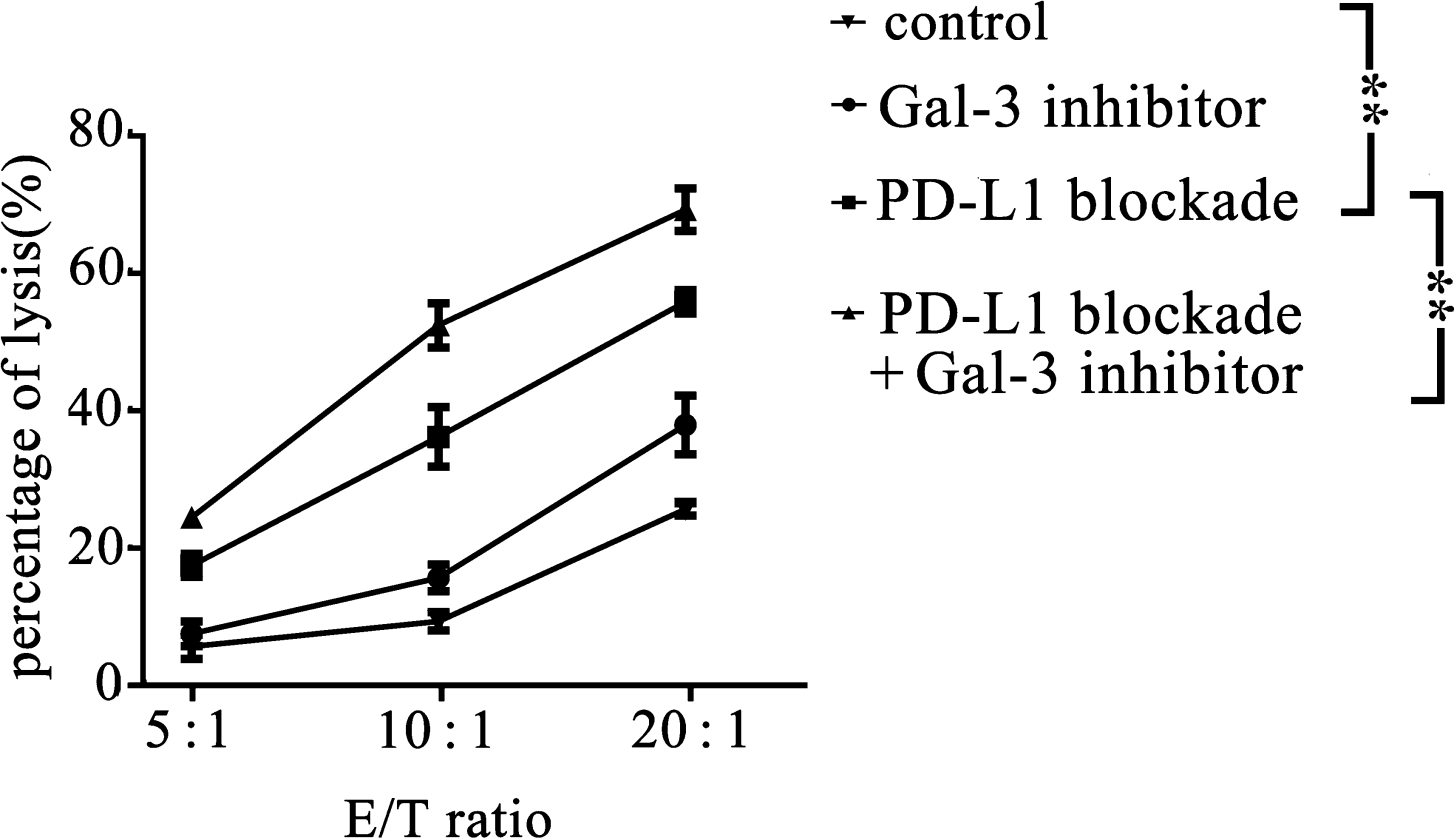
Cytotoxicity assay of T cells. PBMCs were treated with PD‐L1 blockade, Gal‐3 inhibitor, or PD‐L1 blockade + Gal‐3 inhibitor or PBS (control). The targets (A549 cells) were mixed with PBMCs at different E:T ratios, and cytotoxicity was evaluated using a standard 4‐h 51Cr release assay, as described in the Materials and Methods. The data showed that the Gal‐3 inhibitor could enhance the cytotoxic effect of T cells against A549 cells (*P* = 0.000, PD‐L1 blockade VS. control; *P* = 0.000, PD‐L1 blockade VS. PD‐L1 blockade + Gal‐3 inhibitor). All experiments were performed in triplicate, and the data are shown as the mean ± SEM. (*P < 0.05; **P < 0.01, by one‐way ANOVA).

We assessed whether inhibition of Gal‐3 could facilitate the cytotoxic effect of PBMC‐derived T cells induced by PD‐L1 blockade. Cytotoxic activity against A549 cells was assessed using a cytotoxicity assay, and the data showed that coadministration of a Gal‐3 inhibitor promoted the cytotoxic activity of PBMC‐derived T cells induced by PD‐L1 blockade (Fig. 3).

### PD‐L1 blockade in combination with Gal‐3 inhibition induced a synergistic antitumor effect

**Fig. 4:**
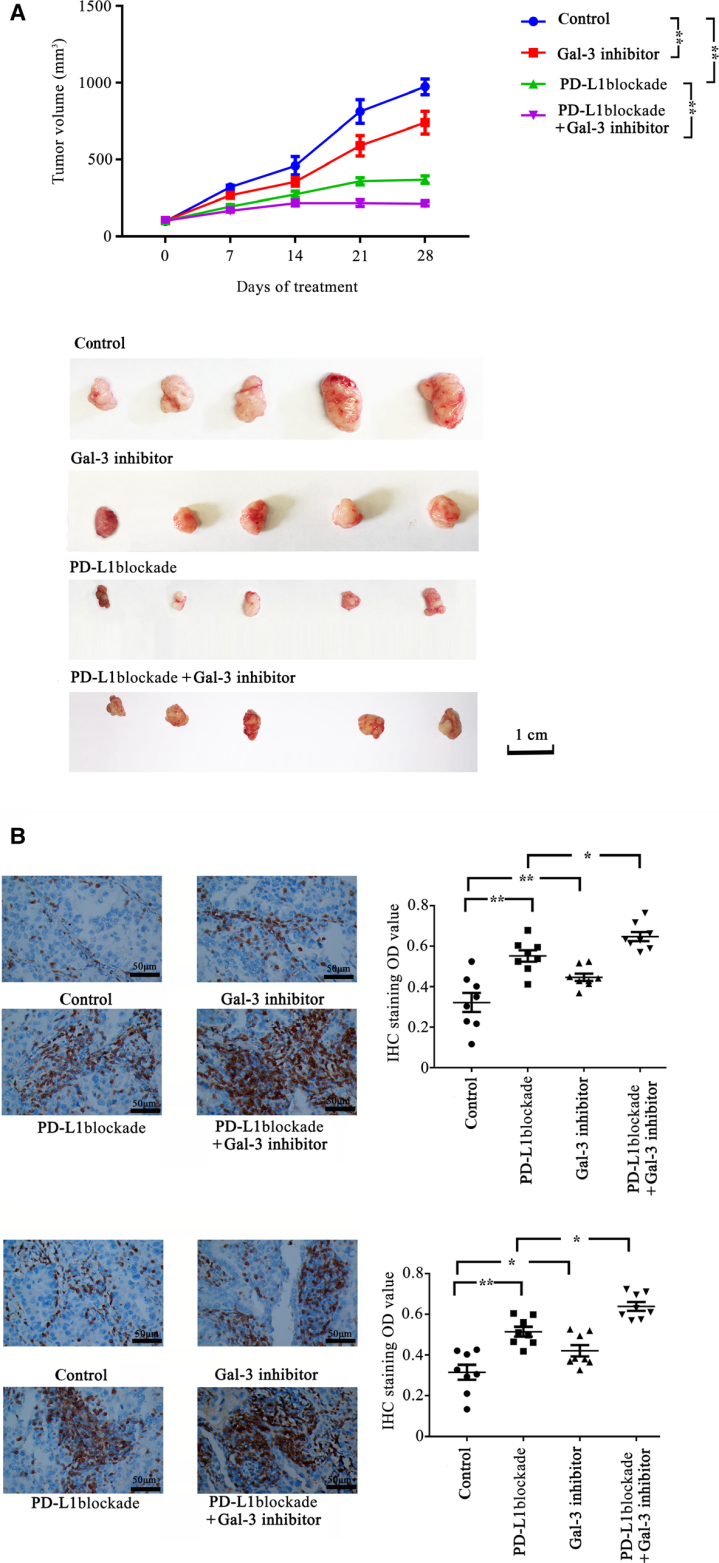
Synergistic antitumor effect of PD‐L1 blockade and Gal‐3 inhibition on tumor growth. BALB/C nude mice were injected with A549 lung cancer cells. When tumor volumes reached 100 mm^3^, all mice received a tail vein injection of PBMCs (1 × 10^7^ cells) on days 1 and 8; the mice (eight mice/group) also received different treatments: PD‐L1 blockade (200 μg, twice per week), Gal‐3 inhibitor (10 mg·kg^−1^·day^−1^), PBS (control). (A) Tumor growth was monitored, and tumor growth curves were generated based on the tumor volume. The data showed that treatment with PD‐L1 blockade (*P* = 0.000) or Gal‐3 inhibitor (*P* = 0.026) alone can significantly inhibit tumor growth compared with the control. The combination of a Gal‐3 inhibitor and PD‐L1 blockade led to a synergistic antitumor effect (the tumor growth inhibition rate of the combination group was 0.21, and *P* = 0.000, PD‐L1 blockade vs. PD‐L1 blockade + Gal‐3 inhibitor). Representative images of xenograft tumors are shown in figure. Scale bar, 1 cm. (B) Immunohistochemistry for CD3 and granzyme B in xenograft tumors. The mouse xenograft experiments were performed as described in the Materials and methods. Combined therapy significantly increased the number of CD3+ TILs (*P* = 0.015, PD‐L1 blockade vs. PD‐L1 blockade + Gal‐3 inhibitor) and granzyme B‐positive cells (*P* = 0.021, PD‐L1 blockade vs. PD‐L1 blockade + Gal‐3 inhibitor) compared with the monotherapy groups. Scale bars, 50 μm. Data are shown as the mean ± SEM. (*P < 0.05, **P < 0.01 by two‐way ANOVA, or by two‐tailed *t*‐test).

We established a mouse xenograft model by the s.c. injection of A549 cells into mice, which were then treated with PD‐L1 blockade, a Gal‐3 inhibitor, or PD‐L1 blockade and a Gal‐3 inhibitor, as described in the methods. Then, tumor growth was monitored (Figs 4A and in Fig. S2). Our results showed that inhibition of Gal‐3 decreased tumor growth compared with the placebo treatment. Moreover, our results indicated a synergistic effect of the combination of PD‐1 blockade with Gal‐3 inhibition (the CI was 0.21). After 28 days of treatment, PD‐L1 blockade resulted in a 62.02% inhibition of tumor growth, while treatment with the Gal‐3 inhibitor resulted in a 23.94% inhibition of tumor growth. In contrast, the combination of PD‐L1 blockade with a Gal‐3 inhibitor induced a 78.17% inhibition of tumor growth.

To further assess whether the Gal‐3 inhibitor enhanced the anti‐PD‐L1 antibody‐mediated promotion of T‐cell cytotoxic activity, we evaluated tumor‐infiltrating lymphocytes (TILs) and their relative activation in tumor tissues derived from mice. The data showed that the combined therapy significantly increased the number of CD3+ TILs. Moreover, in the tumors derived from mice that received combination therapy, the number of cells with granzyme B‐positive expression was significantly higher than that in the mice treated with monotherapy (Fig. 4B).

## Discussion

In the present study, we report that Gal‐3 promoted PD‐L1 expression in lung cancer cells via the STAT3 pathway. The data showed that a Gal‐3 inhibitor, which acted as an effective adjuvant along with PD‐L1 blockade, facilitated the increase in TIL numbers and activity. Moreover, the combination of PD‐L1 blockade and inhibition of Gal‐3 synergistically enhanced the antitumor effect.

Multiple clinical trials have demonstrated the safety and efficacy of PD‐1/PD‐L1 blockade strategies in lung cancer, and the adverse‐event profiles of these strategies do not appear to preclude their use. A phase 2, single‐arm, multicenter trial involving NSCLC patients demonstrated the safety of the first FDA‐approved ICI targeting PD‐1/PD‐L1 (nivolumab), in which grade 3–4 manageable adverse events were observed in 17% of 117 patients [26]. In a pooled analysis of nivolumab, the data showed that the 2‐year overall survival rates in the nivolumab treatment group were superior to those in the docetaxel group [4]. In another study, researchers demonstrated that pembrolizumab improved the rates of overall survival, progression‐free survival, and overall response compared with chemotherapy [27]. However, several other clinical trials found that only ~ 20% of NSCLC patients benefited from ICIs when they were used as a monotherapy [28]. Further research revealed that immunosuppression, such as T‐cell exclusion, induced by cancer cells could limit the efficiency of PD‐1/PD‐L1 blockade. Activation of a plethora of intrinsic and extrinsic immunosuppressive mechanisms can establish an immune‐tolerant microenvironment in tumors [29]. For example, cancer cells can secrete various immunoregulatory cytokines that regulate T cells and establish an immunosuppressive environment [30].

Gal‐3 is highly overexpressed and secreted into the microenvironment by lung cancer cells. NSCLC patients whose tumors had low Gal‐3 expression exhibited an earlier response to pembrolizumab and less disease progression than patients with high Gal‐3‐expressing tumors [31]. Thus, Gal‐3 could have a regulatory effect on PD‐1/PD‐L1 blockade. It is known that hypoxia promotes aggressive phenotype transformation of lung cancer cells whit high proliferation, migration, and therapy resistance. In the present study, we found that hypoxia promoted the expression of Gal‐3 in lung cancer cells. Thus, increased Gal‐3 expression could contribute to lung cancer progression and could be related to a poor prognosis [32]. Our study suggested that Gal‐3 increased PD‐L1 expression in lung cancer cells. Moreover, the regulatory effect of Gal‐3 on PD‐L1 expression might occur through phosphorylation of components of the STAT3 pathway [33]. This phosphorylation enables the activated STAT3 pathway to drive PD‐L1 expression in cancers [34, 35]. Coadministration of a Gal‐3 inhibitor decreased the expression of PD‐1 ligands, and thus, this inhibitor synergistically augmented the effect of PD‐1/PD‐L1 blockade. Capalbo et al demonstrated that low expression of Gal‐3 in tumor cells may result in an early and durable objective response to pembrolizumab compared with that in cases of high Gal‐3 expression [31]. Consistently, Lynda Vuong *et al*. suggested that Gal‐3 inhibition combined with PD‐L1 blockade can generate a synergistic suppressive effect on tumors. In their study, they found that a Gal‐3 inhibitor enhanced the cytotoxic effect of PD‐L1 blockade and resulted in increased expression levels of cytotoxic effectors, such as IFNγ, granzyme B, perforin‐1, and the pro‐apoptotic protein caspase‐3. Moreover, Gal‐3 knockout in a xenograft mouse model resulted in a slower tumor growth rate [19].

Furthermore, our results indicated that treatment with a Gal‐3 inhibitor promoted T‐cell infiltration in tumors, as determined by the increased numbers of CD3‐positive cells and granzyme B‐positive cells. The effect of Gal‐3 on T‐cell function has been recognized for several years, and Gal‐3 could play an immunosuppressive role in T‐cell function. Yang *et al*. [36] reported that Gal‐3 can induce T‐cell apoptosis. Chen *et al*. [37] found that Gal‐3 can destabilize immunological synapses, inhibit peripheral supramolecular activation clusters, and decrease TCR expression in T cells. Kouo *et al*. [38] suggested that Gal‐3 binds to LAG‐3 and thus suppresses CD8+ T cell effector function. Thus, the Gal‐3 expressed and secreted by lung cancer cells into the microenvironment might disrupt the formation of secretory synapses, cytokine secretion [39], and TCR‐associated signaling functions, which would ultimately restrict the function of TILs [40]. However, Gal‐3 inhibition could enhance T‐cell function. Gal‐3‐deficient mice had higher levels of CD8+ T cell effector function and higher expression of several inflammation‐related genes than mice with normal levels of Gal‐3 [38]. Gal‐3 inhibitors have been shown to enhance T‐cell function by increasing tumor M1 macrophage polarization and CD8+ T cell infiltration [19]. Therefore, in addition to inhibition of PD‐L1 expression, Gal‐3 inhibition could suppress tumor growth through the promotion of effector T‐cell infiltration.

In addition, Gal‐3 may directly promote cancer progression and metastasis through multiple pathways. Kataoka *et al*. [41] reported that hypoxia could increase the expression of Gal‐3 in NSCLC cells and thus contributes to metastasis. In esophageal cancer, Gal‐3 can promote cancer progression through activation of the AKT/ERK pathway [42], while Yao *et al*. [43] reported that, in pancreatic cancer, inhibition of Gal‐3 blocked the AKT/FOXO3 signaling pathway and subsequently suppressed cancer progression. Therefore, Gal‐3 promoted lung cancer cell proliferation and migration, whereas blocking Gal‐3 inhibited tumor growth, as was observed in this study. However, few studies have focused on the direct effect of Gal‐3 in lung cancer cells, and thus, additional studies are needed in the future.

## Conclusions

The results of our study provide evidence that Gal‐3 promotes PD‐L1 expression via regulation of the STAT3 pathway. Thus, Gal‐3 inhibitors might augment the efficacy of PD‐L1 blockade against lung cancer and may serve as an effective adjuvant. Combined treatment with Gal‐3 inhibitors and PD‐1/PD‐L1 blockade could be a promising strategy for lung cancer therapy.

## Conflict of interest

The authors declare no conflict of interest.

## Author contributions

HZ, PL, and JC conceived and designed the project. HZ, PL, and YZ completed mainly the experiments. HZ, YZ, LH acquired the data. HZ, JC, ZH, and ZC analyzed and interpreted the data. Both HZ and JC funded this project and wrote the paper.

## Supporting information


**Fig. S1**. IC50 value of Gal‐3 inhibitor in the cells. The cells were seeded in 96‐well plates (5,000 cells per well) and treated with different concentrations of Gal‐3 inhibitor (GB1107) for 72 h. To determine IC50 value of the GB1107, the cells were treated with different concentrations of each drug as follows: 200μM‐0.01μM(with 4 fold interval). Thereafter, cell viability was measured via an MTT assay (Beyotime Biotech, Shanghai, China) in accordance with the manufacturer’s instructions. Absorbance was measured at 440 nm with a multimode plate reader. The data were then used to determine the IC50 values. All experiments were performed in triplicate, and the data are presented as the mean ± SEM.
**Fig. S2**. Representative images of xenograft mice.

## Data Availability

Data will be available from the corresponding author upon reasonable request.
