# Supplementary material for: Inhibition of galectin‐3 augments the antitumor efficacy of PD‐L1 blockade in non‐small‐cell lung cancer
Source: FEBS Open Bio. 2021 Jan 31;11(3):911–20. doi: 10.1002/2211-5463.13088 (PMC7931229; doi:10.1002/2211-5463.13088)

**Figure S1** IC50 value of Gal-3 inhibitor in the cells. The cells were seeded in 96-well plates (5,000 cells per well) and treated with different concentrations of Gal-3 inhibitor (GB1107) for 72 h. To determine IC50 value of the GB1107, the cells were treated with different concentrations of each drug as follows: 200μM-0.01μM(with 4 fold interval). Thereafter, cell viability was measured via an MTT assay (Beyotime Biotech, Shanghai, China) in accordance with the manufacturer’s instructions. Absorbance was measured at 440 nm with a multimode plate reader. The data were then used to determine the IC50 values. All experiments were performed in triplicate, and the data are presented as the mean ± SEM.


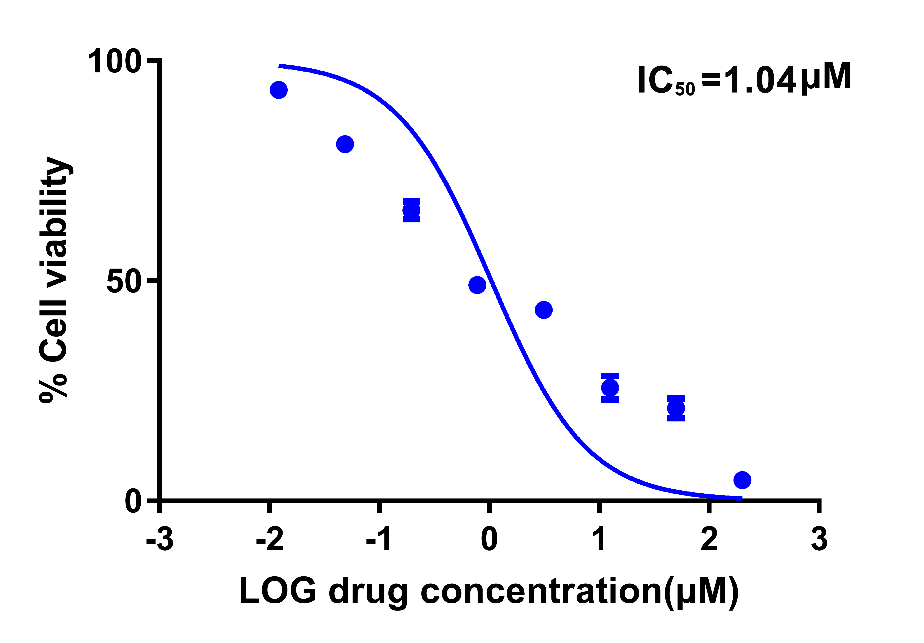


**Figure S2** Representative images of xenograft mice


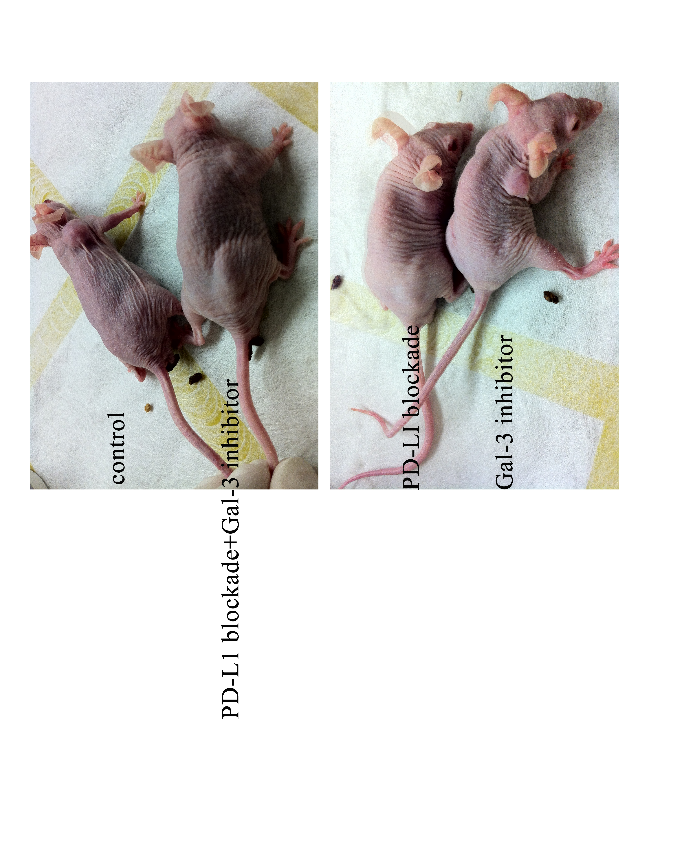

Supplement: Supplementary file 1 — Fig. S1. IC50 value of Gal‐3 inhibitor in the cells. The cells were seeded in 96‐well plates (5,000 cells per well) and treated with different concentrations of Gal‐3 inhibitor (GB1107) for 72 h. To determine IC50 value of the GB1107, the cells were treated with different concentrations of each drug as follows: 200μM‐0.01μM(with 4 fold interval). Thereafter, cell viability was measured via an MTT assay (Beyotime Biotech, Shanghai, China) in accordance with the manufacturer’s instructions. Absorbance was measured at 440 nm with a multimode plate reader. The data were then used to determine the IC50 values. All experiments were performed in triplicate, and the data are presented as the mean ± SEM. Fig. S2. Representative images of xenograft mice. [file FEB4-11-911-s001.docx]
